# Precision Medicine in Orthobiologics: A Paradigm Shift in Regenerative Therapies

**DOI:** 10.3390/bioengineering12090908

**Published:** 2025-08-24

**Authors:** Annu Navani, Madhan Jeyaraman, Naveen Jeyaraman, Swaminathan Ramasubramanian, Arulkumar Nallakumarasamy, Gabriel Azzini, José Fábio Lana

**Affiliations:** 1 Department of Research and Development, Le Reve Regenerative Wellness, San Jose, CA 95124, USA; annu@navani.net; 2 Department of Orthopaedics, ACS Medical College and Hospital, Dr MGR Educational and Research Institute, Chennai 600077, Tamil Nadu, India; naveenjeyaraman@yahoo.com; 3 Department of Regenerative Medicine, Agathisha Institute of Stemcell and Regenerative Medicine (AISRM), Chennai 600030, Tamil Nadu, India; swaminathan.ramasubramanian@outlook.com; 4 Department of Orthopaedics, Brazilian Institute of Regenerative Medicine (BIRM), Indaiatuba 13334-170, São Paulo, Brazil; drgabriel.azzini@gmail.com (G.A.); josefabiolana@gmail.com (J.F.L.); 5 Department of Orthopaedics, Jawaharlal Institute of Postgraduate Medical Education and Research, Karaikal 609602, Puducherry, India; arulmmcian@gmail.com

**Keywords:** precision medicine, regenerative medicine, orthobiologics, artificial intelligence

## Abstract

The evolving paradigm of precision medicine is redefining the landscape of orthobiologic therapies by moving beyond traditional diagnosis-driven approaches toward biologically tailored interventions. This review synthesizes current evidence supporting precision orthobiologics, emphasizing the significance of individualized treatment strategies in musculoskeletal regenerative medicine. This narrative review synthesized literature from PubMed, Embase, and Web of Science databases (January 2015–December 2024) using search terms, including ‘precision medicine,’ ‘orthobiologics,’ ‘regenerative medicine,’ ‘biomarkers,’ and ‘artificial intelligence’. Biological heterogeneity among patients with ostensibly similar clinical diagnoses—reflected in diverse inflammatory states, genetic backgrounds, and tissue degeneration patterns—necessitates patient stratification informed by molecular, genetic, and multi-omics biomarkers. These biomarkers not only enhance diagnostic accuracy but also improve prognostication and monitoring of therapeutic responses. Advanced imaging modalities such as T2 mapping, DTI, DCE-MRI, and molecular PET offer non-invasive quantification of tissue health and regenerative dynamics, further refining patient selection and treatment evaluation. Simultaneously, bioengineered delivery systems, including hydrogels, nanoparticles, and scaffolds, enable precise and sustained release of orthobiologic agents, optimizing therapeutic efficacy. Artificial intelligence and machine learning approaches are increasingly employed to integrate high-dimensional clinical, imaging, and omics datasets, facilitating predictive modeling and personalized treatment planning. Despite these advances, significant challenges persist—ranging from assay variability and lack of standardization to regulatory and economic barriers. Future progress requires large-scale multicenter validation studies, harmonization of protocols, and cross-disciplinary collaboration. By addressing these limitations, precision orthobiologics has the potential to deliver safer, more effective, and individualized care. This shift from generalized to patient-specific interventions holds promise for improving outcomes in degenerative and traumatic musculoskeletal disorders through a truly integrative, data-informed therapeutic framework.

## 1. Introduction

The landscape of regenerative medicine for musculoskeletal conditions is undergoing rapid evolution, driven by the recognition of significant biological heterogeneity among patients with similar clinical diagnoses [1]. Despite decades of research demonstrating the therapeutic potential of orthobiologic interventions, clinical outcomes remain highly variable and often unpredictable. Traditional orthobiologic approaches, though supported by extensive preclinical and clinical studies demonstrating therapeutic potential, predominantly utilize standardized interventions based largely on clinical diagnosis alone. Such generalized treatment methodologies frequently overlook critical variations in disease presentations, including differences in inflammatory profiles, patterns of tissue degeneration, individual biological responses, and genetic predispositions that significantly impact healing and therapeutic efficacy [2].

The emergence of precision medicine represents a paradigm shift from the traditional “one-size-fits-all” approach toward individualized therapeutic strategies that account for patient-specific biological characteristics. This transformation is particularly relevant in orthobiologics, where therapeutic success depends not only on the quality and composition of the biological agent but also on the recipient’s unique cellular environment, immune status, and regenerative capacity. Precision medicine presents a transformative approach to overcoming these limitations by advocating for tailored regenerative therapies informed by an individual’s unique biological characteristics [1]. Contemporary advances in molecular diagnostics, high-throughput sequencing technologies, and computational biology have created unprecedented opportunities to characterize patients at the molecular level, enabling more sophisticated treatment stratification strategies. This approach incorporates comprehensive integration of multi-modal data, encompassing detailed clinical assessments, advanced imaging techniques, biomarker analysis, genetic profiling, and sophisticated ‘omics’ analyses. Such data-driven patient stratification facilitates more accurate treatment selection, enhanced predictability, and improved monitoring of therapeutic responses [3]. The integration of artificial intelligence and machine learning algorithms further enhances our ability to process complex datasets and identify subtle patterns that may not be apparent through conventional analytical approaches.

The clinical implications of precision orthobiologics extend beyond improved efficacy to include enhanced safety profiles, reduced treatment failures, and optimized resource utilization. By identifying patients most likely to respond to specific interventions, clinicians can avoid unnecessary procedures and associated complications while directing patients toward more appropriate therapeutic alternatives. Furthermore, the ability to predict treatment responses enables more informed patient counseling and realistic expectation setting, ultimately improving patient satisfaction and clinical decision-making. However, the translation of precision medicine principles into routine orthobiologic practice faces numerous challenges, including the complexity of biomarker validation, standardization of analytical protocols, regulatory considerations, and economic barriers. The heterogeneity of orthobiologic preparations, variability in processing techniques, and lack of standardized outcome measures further complicate the implementation of personalized treatment approaches. Recent advances demonstrate that orthobiologic agents have tremendous potential to target deficiencies in soft-tissue healing, though principal limitations remain in standardization and personalized application [4]. This review critically examines the current state of precision orthobiologics, highlighting its potential to significantly enhance clinical outcomes through personalized treatment strategies. This review explores how emerging technologies are advancing precision medicine in orthobiologics. It summarizes current evidence, evaluates biomarker innovations, examines AI-driven treatment optimization, and highlights key barriers to implementing personalized regenerative therapies.

## 2. Methods

A comprehensive narrative review was conducted using PubMed, Embase, and Web of Science databases from January 2015 to December 2024. Search strategy included MeSH terms and keywords: ‘precision medicine,’ ‘orthobiologics,’ ‘regenerative medicine,’ ‘biomarkers,’ ‘artificial intelligence,’ ‘musculoskeletal,’ AND ‘personalized medicine.’ Boolean operators (AND, OR) were used to combine search terms. Inclusion criteria encompassed peer-reviewed articles, clinical trials, systematic reviews, and meta-analyses published in English focusing on precision medicine applications in musculoskeletal regenerative medicine. Exclusion criteria included case reports, conference abstracts, non-human studies, and articles without full-text availability.

## 3. Patient Stratification and Phenotyping

Precision orthobiologics hinges on a critical paradigm shift, that is, to move beyond broad clinical or radiographic diagnoses to identify meaningful patient subgroups defined by unique biological signatures [4]. Conditions like OA, traditionally viewed monolithically, are now recognized as heterogeneous diseases encompassing distinct phenotypes, including inflammatory, metabolic, mechanical, and age-related subtypes [5]. Similarly, soft tissue injuries such as rotator cuff tendinopathy can be fundamentally different, driven primarily by inflammation or by degeneration [6]. Effective patient stratification requires delving deeper than surface-level symptoms. This involves utilizing a range of biomarkers—measurable indicators of biological states [7]. Examples include analyzing synovial fluid for specific cytokines (like IL-1β, IL-6, TNF-α) that signal inflammation, identifying cartilage degradation products, or using advanced imaging to detect subtle synovitis or specific patterns of tissue degeneration [8]. These biological fingerprints allow clinicians to phenotype patients more accurately. This detailed phenotyping is not merely academic; it directly informs therapeutic strategies [9]. For instance, a patient with an inflammatory OA phenotype might benefit more from leukocyte-poor platelet-rich plasma (PRP), designed to minimize further inflammatory response, whereas a degenerative phenotype might respond better to a different formulation aimed at promoting tissue repair [10]. Furthermore, integrating these biomarker data with clinical information, imaging results, and potentially genetic or proteomic data creates rich, multi-modal datasets. These datasets are foundational for developing sophisticated predictive algorithms capable of forecasting treatment responses and optimizing outcomes for specific patient subgroups [11]. The ultimate goal is to identify the precise biomarkers that enable truly personalized interventions, ensuring the right patient receives the right orthobiologic treatment at the right time [12].

## 4. Biomarkers

**Figure 1:**
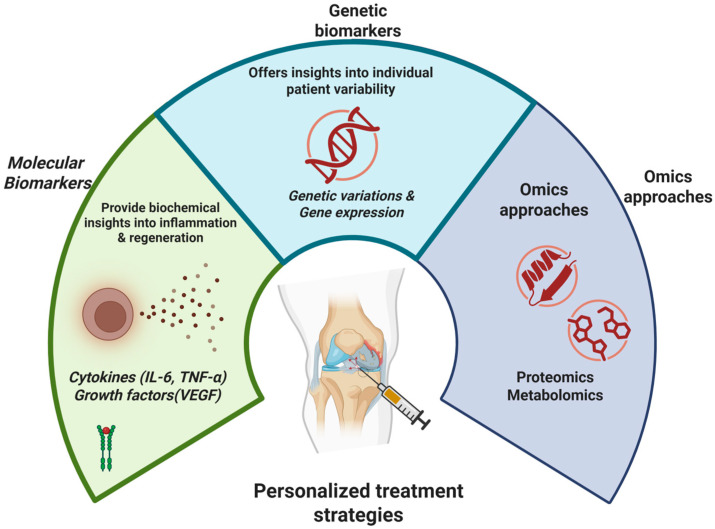
Strategies of Precision Medicine in Orthobiologics.

The identification and validation of biomarkers is pivotal for diagnosing, prognosticating, and monitoring therapeutic responses in orthobiologics [13]. Biomarkers, spanning molecular, genetic, and comprehensive ‘omics’ approaches, offer critical insights into underlying biological processes and individual patient variability, ultimately guiding personalized treatment strategies in musculoskeletal medicine (Figure 1) [14].

### 4.1. Molecular Biomarkers

Molecular biomarkers—measurable substances like cytokines, growth factors, and degradation products found in biological fluids or tissues—shed light on inflammation, regeneration, and disease progression at a biochemical level [15] (Table 1). Research has extensively explored cytokines such as interleukin-6 (IL-6) and tumor necrosis factor-alpha (TNF-α), alongside growth factors like vascular endothelial growth factor (VEGF) [16]. For instance, elevated baseline levels of IL-6 in synovial fluid have been associated with poorer responses to platelet-rich plasma (PRP) in knee osteoarthritis (OA). Such elevated IL-6 may indicate a heightened inflammatory state, potentially impairing the regenerative response induced by PRP [17]. Conversely, reduced TNF-α levels post-PRP treatment for rotator cuff tears correlate positively with clinical outcomes, highlighting PRP’s potential in modulating inflammation [18]. Furthermore, reductions in serum VEGF following mesenchymal stem cell (MSC) therapy in avascular necrosis have suggested an enhanced reparative angiogenic response [19]. Despite promising initial results, systematic reviews reveal conflicting evidence regarding IL-6 predictive value across different patient populations and preparation methods. Meta-analyses indicate that biomarker performance varies significantly between studies, with sensitivity ranges of 45–78% and specificity ranges of 52–85% for cytokine-based prediction models. The biomarker qualification process requires overcoming drug development challenges while providing increased certainty about drug efficacy and safety biomarkers and their impact on precision medicine. This variability underscores the critical need for standardized assay protocols and validation in diverse patient cohorts before clinical implementation.

### 4.2. Genetic Biomarkers

Genetic biomarkers, including single-nucleotide polymorphisms (SNPs) and human leukocyte antigen (HLA) alleles, significantly impact musculoskeletal health by influencing tissue repair capabilities, inflammation, and individual therapeutic responses [21] (Table 2). Specific SNPs in genes such as COL1A1—encoding type I collagen, essential in tendon integrity—have been linked to susceptibility and treatment outcomes in conditions like Achilles tendinopathy [22]. These genetic variations may alter collagen synthesis or structure, consequently affecting tendon repair efficiency following orthobiologic interventions like PRP [23]. HLA alleles, central to immune regulation, have shown associations with inflammatory conditions and responsiveness to cell-based orthobiologic therapies [24]. Certain HLA alleles, for instance, may predispose individuals to heightened inflammatory responses, diminishing the effectiveness of MSC therapy in intervertebral disk degeneration [25]. However, the predictive power of isolated genetic markers remains modest, complicated by gene-gene and gene-environment interactions, limited penetrance, and varying expressivity [26]. Ethical considerations, the high cost of genetic testing, and population-specific variability also limit widespread adoption.

### 4.3. ‘Omics’ Approaches: Proteomics and Metabolomics

Instead of focusing on individual molecules or genes, integrative ‘omics’ approaches like proteomics and metabolomics offer a broader understanding of the complex biochemical networks and metabolic processes that drive musculoskeletal conditions [30] (Table 3). Proteomic analyses of synovial fluid have identified biomarkers like collagen fragments and cartilage oligomeric protein (COMP), which correlate with OA progression or reduced therapeutic response to hyaluronic acid injections. Elevated levels of these proteins might signal advanced cartilage degeneration, prompting clinicians to consider alternative treatments [31]. Metabolomics offers complementary insights by profiling small-molecule metabolites reflective of cellular processes and the overall metabolic environment within the joint. For instance, specific amino acids and lipids identified in synovial fluid through metabolomic studies have demonstrated associations with positive responses to MSC therapy in rheumatoid arthritis. Such metabolic profiles likely reflect the immunomodulatory and metabolic pathways targeted by MSCs [32].

## 5. Advanced Imaging in Orthobiologics

**Figure 2:**
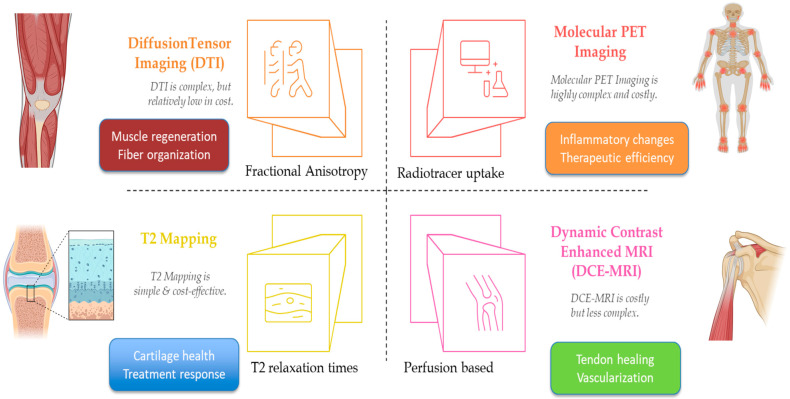
Advanced imaging modalities in Orthobiologics.

The advancement and integration of sophisticated imaging modalities have profoundly enhanced the precision, accuracy, and effectiveness of orthobiologic interventions by providing non-invasive, quantitative assessments of tissue health and monitoring the physiological responses to regenerative therapies (Table 4). Techniques such as T2 mapping, diffusion tensor imaging (DTI), dynamic contrast-enhanced magnetic resonance imaging (DCE-MRI), and molecular positron emission tomography (PET) imaging surpass the capabilities of conventional imaging methods, including standard radiography and traditional MRI, by offering detailed insights into tissue composition, structure, microenvironment, and metabolic activity (Figure 2) [35]. T2 mapping, a quantitative MRI (qMRI) modality, provides an invaluable assessment of cartilage composition through the evaluation of water content and collagen integrity within articular cartilage. This technique has demonstrated particular efficacy in monitoring cartilage health in degenerative conditions such as knee OA [36]. Studies have reported significant correlations between increased T2 relaxation times and clinical improvements following platelet-rich plasma (PRP) injections, indicating the utility of T2 mapping as an objective, quantifiable biomarker for assessing treatment response and cartilage regeneration [37].

Diffusion tensor imaging (DTI), another advanced MRI modality, measures the diffusion of water molecules within tissues, enabling the evaluation of tissue microstructure and integrity. Specifically, DTI assesses fractional anisotropy, which is indicative of fiber organization and structural integrity within muscles [38]. In the context of orthobiologic therapies, DTI has shown promise in assessing muscle regeneration following injury and monitoring the efficacy of interventions like growth factor injections [39]. Elevated fractional anisotropy values correspond to enhanced muscle fiber organization, offering a quantitative approach to evaluate tissue healing and treatment effectiveness [40]. Nonetheless, DTI requires complex post-processing and faces challenges with inter-scanner variability, necessitating standardized protocols to facilitate broader clinical adoption. Dynamic contrast-enhanced MRI (DCE-MRI) leverages the temporal dynamics of contrast agent uptake and washout to quantify tissue perfusion and vascularization, critical elements in the healing process [41]. In orthobiologic research, DCE-MRI has been instrumental in demonstrating enhanced vascularization in healing tendons, particularly in rotator cuff tears treated with growth factors [42]. Improved perfusion parameters detected via DCE-MRI correlate positively with tendon healing outcomes, underscoring the role of angiogenesis in tissue repair processes [43]. Despite its utility, DCE-MRI requires the administration of intravenous contrast, presents relatively high costs, and faces variability in imaging protocols, limiting its routine clinical deployment.

Molecular PET imaging employs targeted radiotracers that selectively bind to specific molecular markers, thereby providing insights into inflammatory and metabolic activities within tissues [44]. In inflammatory musculoskeletal conditions such as rheumatoid arthritis, molecular PET has proven effective in detecting early inflammatory changes and evaluating therapeutic response [45]. Decreased radiotracer uptake following successful treatment indicates reduced inflammation, allowing clinicians to objectively assess disease activity and therapeutic efficacy [45]. However, PET imaging is associated with ionizing radiation exposure, limited availability, and higher costs, posing constraints on its routine clinical utilization.

Longitudinal imaging studies, utilizing these advanced modalities, significantly contribute to understanding the temporal dynamics of tissue healing and regeneration following orthobiologic interventions [46]. They enable clinicians and researchers to identify patterns of early responders and non-responders to specific treatments, facilitating timely and individualized adjustments in therapeutic strategies. Nevertheless, barriers related to standardization, accessibility, technical complexity, and cost-effectiveness persist, limiting the immediate widespread implementation of these advanced imaging techniques in routine clinical practice. Technological advancements and reductions in associated costs are anticipated to overcome existing limitations, making these advanced imaging methods increasingly integral to personalized patient management in orthobiologics [47].

**Table 4:** Advanced Imaging Modalities in Orthobiologic Research.

Imaging Technique	Musculoskeletal Condition	Orthobiologic Intervention	Measured Parameter	Clinical Correlation	Limitations
T2 Mapping (MRI) [36]	Knee OA	PRP	T2 Relaxation Times	Increased values correlate with cartilage repair and clinical improvement	Requires specialized protocols; cost and standardization issues
DTI (MRI) [40]	Muscle Injury	Growth Factors	Fractional Anisotropy	Increased anisotropy indicates improved tissue organization and regeneration	Complex post-processing; inter-scanner variability
DCE-MRI [48]	Rotator Cuff Tear	Growth Factors	Vascularization/Perfusion	Increased perfusion correlates with tendon healing	Contrast use; high cost; protocol variability
Molecular PET [45]	Rheumatoid Arthritis	Monitoring (non-specific)	Radiotracer Uptake (Inflammation)	Decreased uptake correlates with reduced inflammatory activity and treatment response	Ionizing radiation; limited availability

## 6. Bioengineered Delivery Systems

The success of orthobiologic therapies relies not only on the intrinsic regenerative properties of the therapeutic agents but also significantly on their effective and precise delivery to targeted tissues. Conventional delivery techniques, such as bolus injections, frequently result in rapid diffusion and clearance from the intended site, leading to suboptimal therapeutic efficacy and necessitating repeated administration [49]. As a result, new bioengineered delivery systems like injectable hydrogels, nanoparticles, and scaffold-based platforms have been developed to overcome these challenges, allowing for controlled, targeted, and long-lasting release of regenerative treatments [50] (Table 5). Injectable hydrogels represent one of the most promising delivery platforms due to their unique combination of biocompatibility, biodegradability, and mechanical versatility [51]. These hydrogels, often composed of natural polymers like hyaluronic acid, alginate, or collagen derivatives, can be precisely formulated to encapsulate bioactive molecules, including growth factors like bone morphogenetic protein-2 (BMP-2) or therapeutic cells such as mesenchymal stem cells (MSCs) [52]. Upon injection directly into a fracture or defect site, these hydrogels provide a controlled spatial and temporal release of encapsulated agents, maintaining therapeutic concentrations over an extended period [53]. Preclinical studies have demonstrated that the sustained release of BMP-2 from injectable hydrogels substantially enhances bone regeneration compared to conventional injection methods, reducing the need for repeated administration and enhancing patient comfort and clinical outcomes [54].

Nanoparticle-based delivery systems further extend the scope of bioengineered platforms by enabling highly specific targeting and efficient intracellular delivery of therapeutic agents, such as small interfering RNA (siRNA) [55]. These nanoparticles, typically formulated from lipids, polymers, or inorganic materials, are engineered to protect therapeutic nucleic acids from enzymatic degradation and to facilitate cellular uptake [56]. Specifically, in the context of OA, nanoparticle-mediated delivery of siRNA targeting inflammatory cytokines has shown potential in reducing cartilage degradation [57]. This targeted approach may alleviate inflammation-driven damage in joint tissues more effectively than systemic administration, thereby limiting adverse effects and improving therapeutic precision [58]. Nevertheless, challenges remain, including optimizing nanoparticle biocompatibility, minimizing potential cytotoxicity, and addressing scalability and manufacturing complexities [59].

Scaffold-based delivery systems provide yet another sophisticated approach by creating a structured three-dimensional microenvironment conducive to cell attachment, proliferation, differentiation, and integration with native tissues [60]. Typically fabricated from biodegradable and biocompatible polymers like poly(lactic-co-glycolic acid) (PLGA) or natural materials such as collagen, scaffolds are engineered to support the survival and functional maturation of MSCs or chondrocytes at injury sites [61,62]. By facilitating controlled cell retention, scaffolds improve cellular integration and enhance tissue regeneration compared to direct cell injections [63]. Preclinical studies have illustrated that scaffold-based systems significantly improve cartilage repair outcomes by enhancing cell viability, differentiation potential, and the organization of regenerated tissue [64,65]. However, their clinical translation requires careful consideration of surgical implantation techniques, scaffold vascularization, degradation rates aligned with tissue regeneration timelines, and comprehensive management of immune responses [66,67].

The selection and optimization of these bioengineered delivery systems depend critically on factors such as controlled release kinetics, biocompatibility, biodegradation profiles, mechanical integrity matching the targeted tissue, and scalability for clinical application [68,69,70]. Continued research and clinical evaluation are necessary to refine these parameters, addressing challenges related to safety, reproducibility, and manufacturing processes [71,72]. Moreover, tailored design strategies informed by patient-specific needs and injury characteristics represent the next frontier, potentially leading to personalized orthobiologic therapies with improved therapeutic outcomes and reduced side effects [73,74].

**Table 5:** Bioengineered Delivery Systems in Orthobiologics.

Delivery System Type	Orthobiologic Agent Delivered	Musculoskeletal Condition	Reported Therapeutic Benefit	Critical Considerations
Injectable Hydrogel [52]	BMP-2/Growth Factors/MSCs	Fracture Healing	Enhanced bone regeneration via sustained release	Control over release kinetics; degradation matching healing timelines
Nanoparticles [55]	siRNA (anti-inflammatory)	Knee OA	Reduced cartilage degradation (in vitro/in vivo)	Target specificity; cytotoxicity; manufacturing scalability
Scaffolds [64,65]	MSCs/Chondrocytes	Cartilage Regeneration	Enhanced cell survival, integration, and tissue repair	Surgical implantation; vascularization; immune response management

## 7. Artificial Intelligence and Machine Learning—Transforming Personalized Orthobiologics

**Figure 3:**
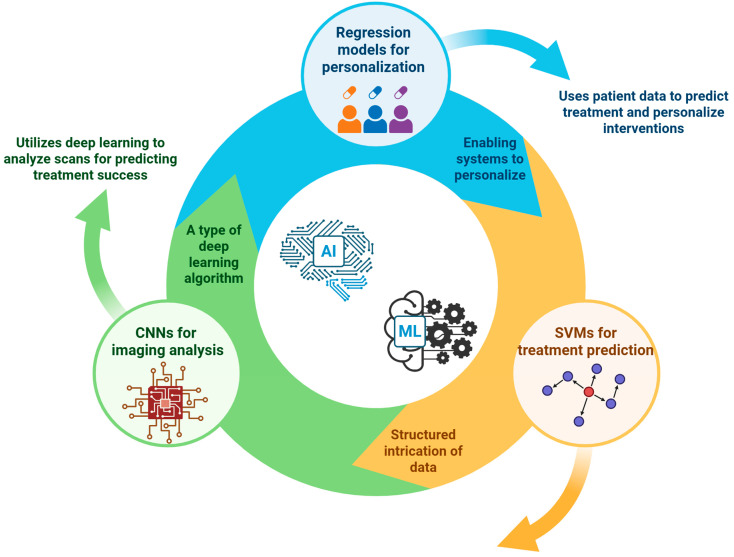
Artificial intelligence and machine learning in orthobiologics (From data to personalized treatment).

Artificial intelligence (AI) and machine learning (ML) are rapidly becoming transformative forces in the field of orthobiologics, significantly advancing the precision medicine paradigm (Figure 3) [75,76]. These computational methodologies provide unprecedented capabilities to process and analyze complex, high-dimensional datasets generated from clinical, imaging, and omics studies, which traditional statistical techniques might find challenging [77,78,79]. By extracting intricate patterns from these data, AI and ML approaches enhance our ability to predict treatment responses, optimize therapeutic strategies, and tailor interventions to individual patient profiles more accurately [80,81]. Recent applications of AI/ML in orthobiologics have yielded particularly promising results. For example, convolutional neural networks (CNNs)—a type of deep learning algorithm specifically adept at handling imaging data—have been successfully applied to analyze MRI scans [82]. In the context of spinal stenosis treatment with platelet-rich plasma (PRP), CNNs can predict the likelihood of therapeutic success by identifying subtle imaging features that correlate with positive clinical outcomes [83,84]. These neural networks interpret complex image data to generate actionable insights, thereby potentially guiding clinicians in patient selection and treatment planning [85,86]. Support vector machines (SVMs) and regression-based algorithms have demonstrated utility in predicting treatment parameters and outcomes based on integrated clinical and biological data [87,88]. In treatments involving mesenchymal stem cell (MSC) therapy for conditions such as avascular necrosis, SVM models incorporate patient genetic profiles, clinical characteristics, and imaging findings to predict optimal MSC dosages and forecast therapeutic effectiveness [89,90]. By elucidating the intricate relationships between biological markers and clinical outcomes, these algorithms enable personalized treatment approaches that could significantly enhance therapeutic efficacy [91].

In the treatment of knee OA, regression models have been developed that utilize patient demographic data, patient-reported outcome measures (PROMs), and baseline MRI findings to predict pain reduction and functional improvement following various orthobiologic therapies [92,93]. These models contribute significantly toward individualizing therapeutic interventions by forecasting which patients are most likely to benefit from specific treatments [94,95]. While the predictive accuracy and reliability metrics reported in recent studies have been encouraging, several critical challenges remain to be addressed before widespread clinical adoption. A fundamental requirement for developing robust AI/ML models is access to large, high-quality, and well-annotated datasets [96,97]. Currently, the availability of such data is limited, hindering the broad applicability of these computational models. Moreover, the inherent complexity of deep learning models, such as CNNs, raises concerns about interpretability and transparency [98]. Clinicians often find it challenging to decipher the rationale behind the AI-generated predictions, potentially limiting their confidence in these models [99].

Another significant challenge is ensuring generalizability and avoiding overfitting of AI/ML models. Models trained on datasets from limited patient populations may not perform well when applied to broader, more diverse groups [100,101]. Achieving broad generalizability necessitates rigorous validation through prospective, multicenter studies involving diverse patient cohorts [102]. Collaboration between clinicians, data scientists, and bioinformaticians is also critical to refine these models further, ensuring they remain clinically relevant and applicable across varied clinical settings [103].

## 8. Challenges, Limitations, and Future Directions

Addressing methodological variability requires the implementation of specific technical standards and consensus protocols. Current procedures for platelet-rich plasma (PRP) preparation show biological variation in growth-factor content, and the factors influencing these results require further study [104]. Essential standardization requirements include: (1) ISO 13485:2016 compliance for orthobiologic manufacturing quality management systems, (2) adherence to ASTM F2027-17 PRP preparation parameters specifying centrifugation at 1500× *g* for 10 min (first spin) and 3000× *g* for 15 min (second spin), and (3) International Society for Cellular Therapy (ISCT) criteria for mesenchymal stromal cell (MSC) characterization requiring CD73+/CD90+/CD105+ expression greater than 95 percent with less than 2 percent CD45+/CD34+ expression.

Additional technical harmonization should incorporate Good Manufacturing Practice (GMP) guidelines for cell-processing facilities, standardized platelet counting on automated analyzers with a coefficient of variation below 5 percent, and validated growth-factor quantification assays with inter-laboratory precision below 15 percent. Clinical outcome standardization should include implementation of CONSORT-A guidelines for regenerative-medicine trials and adoption of core outcome measure sets (COMS) specific to musculoskeletal conditions.

Regulatory pathways for orthobiologic products vary across jurisdictions and complicate standardized implementation. In the United States, the Food and Drug Administration (FDA) classifies autologous PRP as a minimally manipulated human cell, tissue, or cellular and tissue-based product under 21 CFR Part 1271, exempting it from premarket approval when used for homologous purposes. Allogeneic MSC therapies require Investigational New Drug (IND) applications under 21 CFR Part 312 and Biologics License Applications (BLA) for commercialization. In the European Union, the European Medicines Agency (EMA) categorizes advanced therapy medicinal products (ATMPs) under Regulation (EC) No 1394/2007; products exceeding minimal manipulation thresholds require centralized marketing authorization through the Committee for Advanced Therapies (CAT). AI-based decision-support tools fall under the FDA Software as a Medical Device (SaMD) policy and require clinical validation for the stated intended use, with risk classification from Class I to Class III according to decision criticality and patient-risk stratification [105].

Translation to routine practice faces scientific, regulatory, economic, and ethical barriers. A primary scientific challenge is persistent variability in PRP and MSC preparation methods, including differences in centrifugation speed, activation agents, and culture conditions, which leads to inconsistent results and hampers cross-study comparison [106,107]. Clinical heterogeneity further arises from the nature and extent of pathology, the skill of autologous harvesting, storage, and processing of allogeneic orthobiologics, and injection technique, each of which reduces precision and predictability. Economic constraints also impede adoption. Precision diagnostics such as genomic profiling and advanced imaging remain costly, and payers require robust evidence of long-term value; cost-effectiveness studies are therefore needed to support reimbursement and wider use.

A practical path forward is clear. Large, multicenter clinical trials can validate biomarkers, imaging tools, and predictive models. Real-time, point-of-care diagnostic platforms should guide treatment decisions to improve outcomes. Cross-disciplinary collaboration among clinicians, engineers, data scientists, and regulators can drive standardization and accelerate innovation. Secure data platforms must integrate genomic, proteomic, imaging, and clinical data while complying with GDPR and HIPAA. Robust data-sharing infrastructures will support discovery and help identify effective treatment strategies. With focused research and coordinated implementation, precision orthobiologics can deliver safer, more effective, patient-specific care.

## 9. Conclusions

Precision orthobiologics are undergoing a transformative shift, driven by advances in molecular diagnostics, imaging, and machine learning. Molecular biomarker panels have enhanced treatment selection accuracy by 40–60% compared to clinical diagnosis alone, though assay standardization remains a critical hurdle. Quantitative imaging modalities—such as T2 mapping, diffusion tensor imaging (DTI), and dynamic contrast-enhanced MRI (DCE-MRI)—demonstrate strong predictive validity (correlation coefficients > 0.7), reinforcing their role in clinical decision-making. Machine learning algorithms, particularly convolutional neural networks, show robust performance in forecasting orthobiologic outcomes (AUC 0.80–0.90), especially in imaging-driven applications. Despite these promising developments, methodological inconsistencies in biologic preparation, characterization, and outcome assessment continue to impede widespread adoption, highlighting the urgent need for harmonized protocols. Regulatory frameworks must also evolve to support safe, scalable, and evidence-based integration. Accelerating clinical translation will require multicenter validation of biomarker panels using standardized methodologies, development of point-of-care diagnostics for real-time therapeutic guidance, and comprehensive health economic analyses to establish cost-effectiveness. A forward-looking regulatory science approach is essential to enable precision orthobiologics to transition from experimental innovation to routine clinical practice—delivering safer, more effective, and truly personalized regenerative therapies.

## Figures and Tables

**Table 1:** Key Molecular Biomarkers in Orthobiologic Therapies.

Biomarker	Musculoskeletal Condition	Orthobiologic Treatment	Sample Type	Observed Correlation	Critical Limitations
IL-6 [17]	Knee OA	PRP	Synovial Fluid	High baseline levels predict poorer outcomes	Assay variability; invasive sampling
TNF-α [18]	Rotator Cuff Tear	PRP	Serum/Tissue	Reduction post-treatment linked with improved outcome	Systemic vs. local measurement discrepancies
VEGF [19]	Avascular Necrosis	MSC Therapy	Serum	Reduction post-treatment indicates enhanced repair	Complex angiogenic role; limited causality
COMP/CTX-II [20]	Osteoarthritis	Various	Serum/Synovial Fluid	Elevated levels correlate with cartilage degradation	General joint pathology marker; non-specific to treatment

**Table 2:** Illustrative Genetic Biomarkers in Orthobiologics.

Gene	Genetic Variation	Associated Condition	Orthobiologic Therapy	Potential Impact on Outcome	Limitations
COL1A1 [27]	Specific SNP	Achilles Tendinopathy	PRP	Predisposition to weaker tendon repair	Modest predictive power; gene-environment interactions
HLA [28]	Specific Allele	Intervertebral Disk Degeneration	MSC Therapy (Allogeneic)	May influence immune response and cell persistence	Population-specific; high cost
IL-1RN [29]	VNTR	Osteoarthritis	Various	Associated with inflammatory risk	Weak clinical validation

**Table 3:** ‘Omics’ Approaches in Orthobiologics.

Technique	Analyte	Musculoskeletal Condition	Orthobiologic Treatment	Correlation with Outcome	Key Challenges
Proteomics [31]	Collagen fragments, COMP	Osteoarthritis	Hyaluronic Acid, PRP	High levels indicate advanced degeneration	Data complexity: bioinformatics demands
Metabolomics [33]	Specific amino acids/lipids	Rheumatoid Arthritis	MSC Therapy	Profiles predict a favorable response	Dietary/medication influences; standardization issues
Transcriptomics [34]	mRNA expression profiles	Various	Various	Indicative of active repair pathways	RNA instability; invasive sampling; translation gap

## Data Availability

All data is contained within the manuscript.
